# Trichomonicidal and parasite membrane damaging activity of bidesmosic saponins from *Manilkara rufula*

**DOI:** 10.1371/journal.pone.0188531

**Published:** 2017-11-30

**Authors:** Patrícia de Brum Vieira, Nícolas Luiz Feijó Silva, Camila Braz Menezes, Márcia Vanusa da Silva, Denise Brentan Silva, Norberto Peporine Lopes, Alexandre José Macedo, Jaume Bastida, Tiana Tasca

**Affiliations:** 1 Laboratório de Pesquisa em Parasitologia, Faculdade de Farmácia, Universidade Federal do Rio Grande do Sul, Porto Alegre, RS, Brasil; 2 Programa de Pós-graduação em Ciências Biológicas, Universidade Federal do Pampa, São Gabriel, RS, Brasil; 3 Centro de Ciências Biológicas e Departamento de Bioquímica, Universidade Federal de Pernambuco, Recife, PE, Brasil; 4 Laboratório de Produtos Naturais e Espectrometria de Massas, Universidade Federal de Mato Grosso do Sul, Campo Grande, MS, Brasil; 5 Núcleo de Pesquisas em Produtos Naturais e Sintéticos, Faculdade de Ciências Farmacêuticas de Ribeirão Preto, Universidade de São Paulo, Ribeirão Preto, SP, Brasil; 6 Faculdade de Farmácia e Centro de Biotecnologia, Universidade Federal do Rio Grande do Sul, Porto Alegre, RS, Brasil; 7 Instituto Nacional do Semi-Árido (INSA), Núcleo de Biprospecção da Caatinga (NBioCaat), Campina Grande, PE, Brasil; 8 Departament de Productes Naturals, Facultat de Farmacia, Universitat de Barcelona, Barcelona, Spain; Universita degli Studi di Parma, ITALY

## Abstract

The infection caused by *Trichomonas vaginalis* is the most common but overlooked non-viral sexually transmitted disease worldwide. Treatment relies on one class of drugs, the 5-nitroimidazoles, but resistance is widespread. New drugs are urgently needed. We reported the effect of crude and purified saponin fractions of *Manilkara rufula* against *Trichomonas vaginalis*. The compound responsible for antitrichomonal activity was isolated and identified as an uncommon bidesmosic saponin, Mi-saponin C. This saponin eliminated parasite viability without toxicity against the human vaginal epithelial line (HMVII). In addition, the isolated saponin fraction improved the metronidazole effect against a metronidazole-resistant isolate and dramatically reduced the cytoadherence of *T*. *vaginalis* to human cells. Investigation of the mechanism of death showed that the saponin fraction induced the parasite death due to profound membrane damage, inducing a disturbance of intracellular content without nuclear damage. To the best of our knowledge, this is the first report of antitrichomonal activity in the bidesmosic saponins of *Manilkara rufula*.

## Introduction

*Trichomonas vaginalis* is a parasitic protozoan that occurs in the urogenital tract and is the causative agent of trichomoniasis, which is the most widespread non-viral sexually transmitted disease (STD) in humans. Globally, this infection affects 276 million people per year and increases susceptibility to HIV infection and the risk of cervical and prostate cancer [1]. The mainstay medication for trichomoniasis is metronidazole (MTZ), but, increased cases of resistance are now being observed [2,3]. Therefore, efforts to identify new alternatives to control trichomoniasis are vital.

In this context, natural products are a viable alternative to synthetic drugs. From 1981 to 2014, sixty-five percent of new approved drugs for anti-parasitic treatment were natural products or derivatives [4] demonstrating their huge potential as a new strategy, the includes topical treatment of vaginal infections [5]. The potential of natural products for drug discovery was recently highlighted, which was describing their re-emergence in a new genomics era [6]. Some isolated natural products have demonstrated anti-*T*. *vaginalis* activity, such as alkaloids, saponins, phenolics, terpenoids and polyacetylenes [7]. In addition, a polyherbal vaginal medicine has been formulated with *Azadirachta indica* extract, purified saponins from *Sapindus mukerossi* and *Mentha citrate* oil; this formulation showed a 100% cure rate for *T*. *vaginalis* infection in a phase II trial, as well as for *Candida albicans* (77% of inhibition) and bacterial vaginosis (68% of inhibition) [8], highlighting the capacities of extract applications.

The specie *Manilkara rufula*, also known as “maçaranduba”, belongs to the Sapotaceae family. Several activities have been attributed to the *Manilkara* species, including antimicrobial, anti-parasitic, and anti-cancer properties [9]. The miscellaneous activity demonstrated by the *Manilkara* genus is attributed to the diversity of secondary metabolites present in these species as triterpenoid saponins [10], triterpenes [11], flavonoids [12], and tannins [13]. However, there are few studies regarding *M*. *rufula*, and only one study was found in the literature for the biological effects of this plant [14], demonstrating the potential of this plant as a source of new compounds and biological activities.

Saponins are steroid or triterpene glycosides that form stable soap-like foams in aqueous solution due to of their amphiphilic nature, due to the linkage of isoprenoidal-derived aglycone to one or more sugar moieties. Saponins are also classified as monodesmosidic, bidesmosidic or tridesmosidic, depending on whether one, two or three positions on the aglycone are attached with sugar chains, respectively. They exhibit a wide variety of biological activities, e.g., anti-inflammatory, anti-cancer, anti-bacterial, anti-fungal, antiviral, immunomodulatory, anti-hepatotoxic, molluscicidal, antidiabetic, anti-viral, and anti-trichomonal [15–17]. In addition, monodesmosic and bidesmosic saponins have revealed significant differences in biological activities [17].

Saponins have showen high anti-trichomonas activity, but they are still underexplored, and few studies are reported regarding this application. The monodesmosic saponins from *Hedera colchica* [18] and *Buddleja madagascariensis* [19] exhibited high antitrichomonas activity, as well as the enriched fractions with monodesmosidic saponins from *Passiflora alata* and *Quillaja saponaria*, which were more active than the fraction of bidesmosic saponins from *Ilex brasiliensis* [15]. In addition, the fraction of saponins (monodesmosic and bidesmosic) from *Sapindus* sp. [20] also demonstrated high activity, and thus, the relationship between of the saponin structure and anti-trichomonas activity is not clear.

In this study, crude and purified saponin fractions of *M*. *rufula* were tested against *T*. *vaginalis*, and the mechanism of death was investigated. In addition, constituents were identified using LC-ESI-MS and MALDI-MS, and an uncommon bidesmosic saponin active against trichomonas was isolated and characterized by HRMS and NMR.

## Materials and methods

### General procedure

MALDI-MS and MALDI-MS/MS analyses were performed using UltrafleXtreme MALDI-TOF/TOF equipment (Bruker Daltonics, Bremen, Germany), applied in reflector positive ion mode. Ions were accelerated at 20 kV and reaccelerated to 19 kV in the LIFT cell for MS/MS. Laser frequency was 1000 Hz and pulsed ion extraction was 100 ns. External calibration was conducted using a peptide calibration standard from Bruker Daltonics. As a matrix, 2,5-dihydroxybenzoic acid (DHB) was used at a concentration of 20 mg/mL (4:6 of acetonitrile and water, v/v). A 0.1 M NaCl solution was added to the mixture of matrix and sample solution (10 mg/mL), and after homogenization, 1.0 μL was placed onto a MALDI target. LC-DAD-MS analyses were performed with an UFLC Shimadzu Prominence coupled to a diode array detector (DAD) and a high resolution MicrOTOF-Q III mass spectrometer (Bruker Daltonics, Billerica, MA, USA). A Kinetex C18 column (2.6 μm, 150 × 2.1 mm, Phenomenex) was used. The flow rate was 0.3 mL/min, and 1.0 μL was the injection volume of the samples. The mobile phase was composed of acetonitrile (solvent B) and deionized water (solvent A)-formic acid 0.1% (v/v). The gradient elution profile was: 0–2 min—3% of B, 2–25 min—3–25% of B, 25–40 min—25–80% of B; 40–43 min—80% of B. The analyses were performed at 50°C in negative and positive ion modes. N_2_ was applied as a nebulizer gas (4 Bar) and dry gas (9 L/min). The capillary voltage was 2.5 kV. The H100 fraction was solubilized in acetonitrile and water 1:1 (v/v) at a concentration of 1.0 mg/mL, subsequently the solution was filtered (Millex 0.22 μm, PTFE, Millipore) and injected. The 1D and 2D NMR spectra were acquired on a Bruker DRX500 spectrometer with the deuterated solvent CD_3_OD (δ 3.31) used as an internal standard.

### Materials

HPLC methanol, acetonitrile, trifluoroacetic acid (TFA), formic acid, and ultrapure water (Milli-Q Millipore) were used throughout the study. Metronidazole (total impurities: ≤0.0005% phosphorus and ≤0.1% insoluble matter), 3-(4,5-dimethylthiazol-2-yl)-2,5-diphenyltetrazolium bromide (MTT), RPMI-1640 and Dulbecco's Modified Eagle’s Medium (DMEM) were obtained from Sigma-Aldrich, St. Louis, MO, USA. Sephadex® LH20 was obtained from GE Healthcare Life Sciences. The matrix 2,5-dihydroxybenzoic acid (DHB) was purchased from Bruker Daltonics, and NaCl was obtained from Synth.

### Plant material

The leaves of *M*. *rufula* were harvested at the Parque Nacional do Catimbau (PARNA do Catimbau), Pernambuco, Brazil (8°37’S 37°08’W) in February 2012 with authorization from the Instituto Chico Mendes de Conservação da Biodiversidade (ICMBio) number SISBIO 16.806. A voucher specimen was deposited in the Herbarium of the Instituto Agronômico de Pernambuco (IPA84889) and identified by Alexandre da Silva Gomes.

### Extraction and isolation

The crude extract of *M*. *rufula* was prepared by macerating the leaves with ethanol:water [21], and an enriched saponin fraction (H100) was obtained using a Sephadex^®^ LH20 column eluted with 100% water followed by freeze-drying. A second Sephadex^®^ LH20 column was performed and eluted with water, water:methanol (50:50 and 30:70), methanol, water and acetone. One hundred and thirty fractions (F1-130) were obtained and analyzed by MALDI-MS. The fractions (F23-27) were also analyzed by NMR, and Mi-saponin C (1) was structurally identified.

### Parasite cultivation

Isolates of *T*. *vaginalis* were used in the study: ATCC 30236 (*JH 31A #4)* and fresh clinical isolates: TV-LACM2 (MTZ-sensitive), TV-LACM2R (MTZ-resistant), TV-LACM5, TV-LACM6 (high-adherent), TV-LACM11, TV-LACM22, TV-LACM24, TV-LACH4, and TV-LACH6. All fresh clinical isolates were obtained from Laboratório de Análises Clínicas e Toxicológicas, Faculdade de Farmácia, UFRGS, Brazil, with ethical approval by the Universidade Federal do Rio Grande do Sul (UFRGS) Research Ethical Committee (number 18923). Organisms were cultured in TYM medium as described by Diamond [22]. Only parasites at the logarithmic phase of growth and exhibiting motility and normal morphology were harvested, centrifuged and resuspended in fresh TYM for assays. The parasite density of 1.0 x 10^5^ trophozoites/mL was used throughout the study unless stated otherwise. All experiments were performed in triplicate with at least three independent cultures (n = 3).

### Anti-*Trichomonas vaginalis* assays and minimum inhibitory concentration

The anti-*T*. *vaginalis* activity of H100 and purified fractions (F10, F27, F31, F33, F35 and F113) was performed *in vitro* [23]. Purified fractions were tested at 0.5 mg/mL. Trophozoite viability was evaluated compared to untreated parasites by counting in a hemocytometer using trypan blue dye exclusion. MIC was confirmed by the failure of non-motile parasites to grow after re-inoculation into drug-free medium. MIC was used in the following experiments.

### Kinetic growth curve

The effect of the H100 fraction on parasite growth and viability was evaluated, and a kinetic growth curve was prepared. Parasites were treated or untreated with the H100 fraction at 0.5 mg/mL and incubated for 2, 4, 6, 12, 24, 48, 72, and 96 h. After counting using a hemocytometer, the results were expressed as trophozoite number per milliliter compared to untreated organisms.

### Fluorescein diacetate and propidium iodide viability assay

Parasites were treated or untreated with the H100 fraction at 0.5 mg/mL for 2, 4, 6, 12 and 24 h. The viability of the parasites was assessed using 10 μg/mL fluorescein diacetate (FDA) and 10 μg/mL propidium iodide (PI) for 20 min before fluorescence analyses. Cytometric dot plots were acquired using the flow cytometer FACsVerse.

### Cytotoxicity against human cells

The cytotoxicity of H100 was determined using two cell lineages, HMVII (ECACC 92042701) and HeLa (ATCC CCL-2 from BCRJ 0100) (vaginal and cervical epithelial cells, respectively). The cells were grown and maintained as described previously [23]. Before assay, 3.0 x 10^4^ or 1.0 x 10^4^ cells per well were seeded in 96-well microtiter plates overnight. Then, the medium was replaced with fresh medium containing H100 at 0.5 mg/mL. Negative (medium without H100) and positive controls (0.2% Triton X-100) were added. The cytotoxicity was evaluated after 24 or 48 h of incubation, and cell viability was assessed by the MTT assay.

### H100 and metronidazole synergic effect

To evaluate the synergic effect of H100 and MTZ, the isolate TV-LACM2R was used. The MIC of H100 was determined, and then TV-LACM2R was treated with 0.5 (MIC/2) and 1.0 mg/mL H100 MIC with or without 0.0026 mg/mL (sub-lethal concentration) and 0.012 mg/mL (MIC) MTZ. Microplates were incubated for 24 h at 37°C and 5.0% CO_2_, and the viability was assessed by hemocytometer counting.

### Cytoadherence assays

*Trichomonas vaginalis* (TV-LACM6) stained with CellTracker^®^ was treated with 0.5 mg/mL H100 or 10 mM periodate (positive control). After treatment, stained organisms were added to confluent cells (HeLa and HMVII) and incubated for 30 min at 37°C and at 5% CO_2_. Afterwards, wells were gently washed twice with complete RPMI to remove non-attached parasites. The attached cells were released by trypsinization and quenched with the addition of bovine serum. After being released from the wells, the samples were analyzed by flow cytometry using a FacsVerse cytometer.

### Reactive oxygen species production

To examine whether incubation with H100-treated *T*. *vaginalis* can induce ROS production in human neutrophils, neutrophils were isolated from the heparinized blood of healthy human donors as described in a previous study [24] and co-incubated with or without *T*. *vaginalis* and treated or untreated with H100 at 0.5 mg/mL, as described by Song et al. [25]. ROS generation was evaluated by flow cytometry using a FacsVerse cytometer.

### Hemolytic activity

The effect of H100 on erythrocyte membranes was evaluated as previously described [15]. Briefly, erythrocyte solution (1%) was treated with H100 and incubated for 1, 24 and 48 h at 37°C. The supernatant absorbance was measured at 540 nm, and the hemolysis percentage was calculated compared to a 100% hemolysis control (Triton-X 100 0.2%).

### Scanning and Transmission Electron Microscopy (SEM and TEM)

The effect of the H100 fraction on *T*. *vaginalis* ultrastructure was analyzed using SEM and TEM. Trophozoites were treated or not (control) with H100 at 0.5 mg/mL for 4 or 24 h and them, collected by centrifugation, and fixed for 2 h 30 min with 2.5% glutaraldehyde in 0.1 M sodium cacodylate buffer (pH 7.2). Parasites were post-fixed for 2 h in 1.0% OsO_4_ in 0.1 M sodium cacodylate buffer (pH 7.2) and dehydrated in acetone. The organisms were dried to the critical point using CO_2_ and coated with gold-palladium for SEM visualization [26]. Organisms were embedded in EmBed^®^ 812-DER736 resin and ultrathin sections were cut and stained with uranyl acetate and lead citrate for TEM visualization [27].

### Statistics

Student’s *t* test was used for comparisons between two groups. The results are expressed as the mean ± SD of at least three individual experiments. Statistical significance was *P* ≤ 0.05. Analyses were performed using Statistical Package for the Social Sciences (SPSS) v.14 software.

## Results and discussion

**Fig 1:**
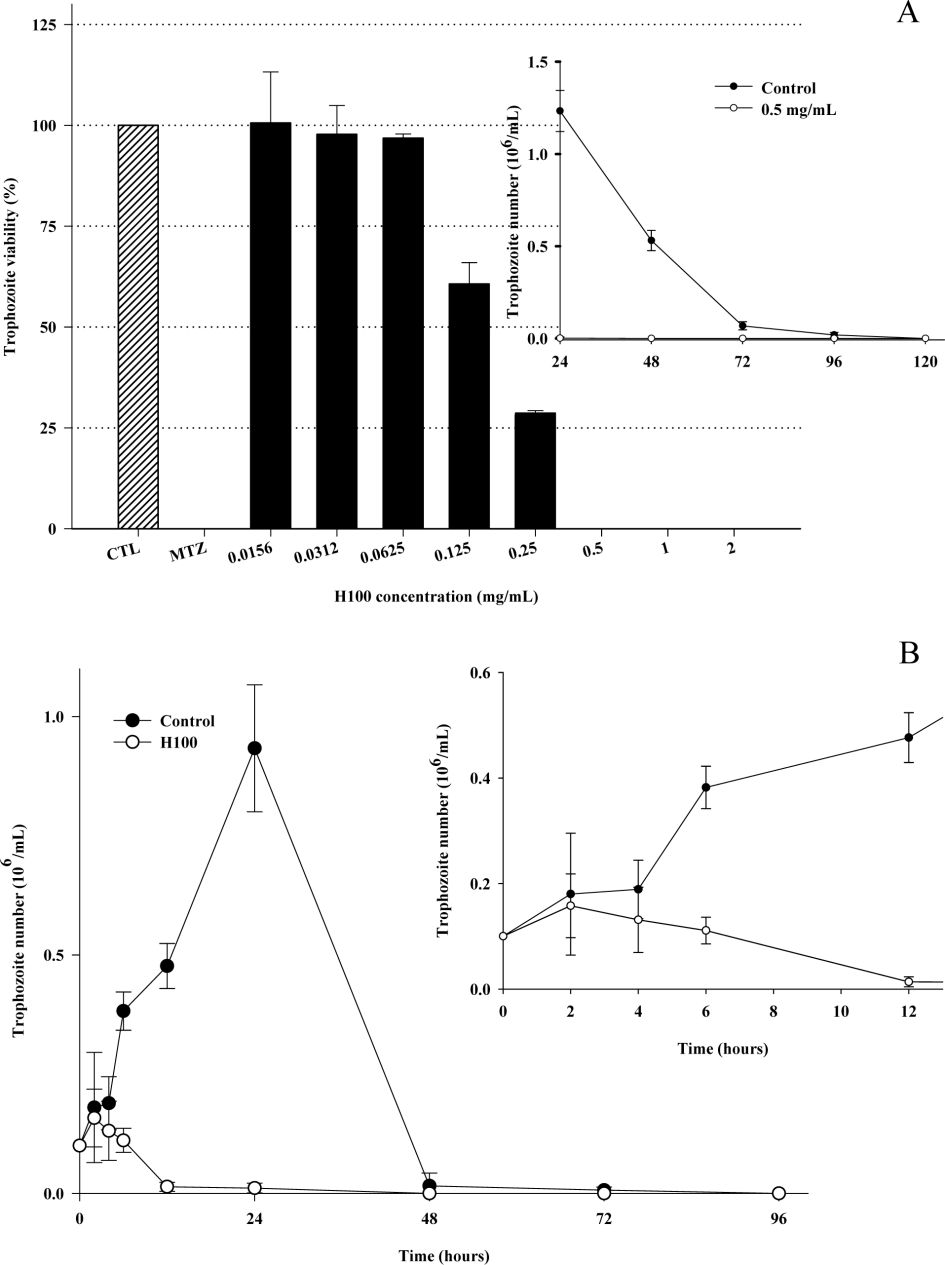
Effect of the H100 fraction against *Trichomonas vaginalis*. MIC determination of the H100 fraction against the *T*. *vaginalis* 30236 isolate (A). Inset: MIC confirmation by counting trophozoite number after incubation on TYM fresh medium; kinetic growth curve of H100 (0.5 mg/mL), treated trophozoite compared to control (untreated parasites) (B). Data are the mean ± SD of at least three different experiments (parasite suspensions) performed in triplicate.

In this study, a potent *in vitro* effect of *M*. *rufula* against the parasite *T*. *vaginalis* was demonstrated with an MIC of the H100 saponin fraction of 0.5 mg/mL (Fig 1A). The MIC was determined by trophozoite counting under microscopic observation with exclusion dye, which is a laborious method. To overcome subjective counting mistakes, parasites at the MIC were inoculated in fresh TYM medium and the viability was assessed every 24 h over 120 h (5 days). After 24 h of incubation in new medium, there was no parasite growth at 0.5 mg/mL H100, confirming the MIC and the trichomonicidal effect, since parasite proliferation was still eliminated in drug-free medium (Fig 1A, inset). The effect of H100 on parasite growth was kinetically determined, and immediately after 4 h of incubation, a reduction in the parasite number was observed. As expected, a total elimination of parasite growth was observed after 24 h, while the well-grown control trophozoites were highest in number at this time point (Fig 1B). In addition, parasite viability was measured using PI-FDA labeling. Organisms with intact membranes retain FDA dye and are assumed to be viable. In turn, trophozoites with a nonfunctional membrane incorporate PI dye. As shown in S1 Fig, untreated organisms presented only FDA labeling. H100-treated *T*. *vaginalis* displayed FDA staining; however, the reduced parasite number was evident and confirmed using cytometric quantification analyses (S2 Fig). Cytometry analysis agreed with the counting assays, indicating that while it is laborious, microscopic counting is a suitable method for *T*. *vaginalis* viability and MIC determination.

**Fig 2:**
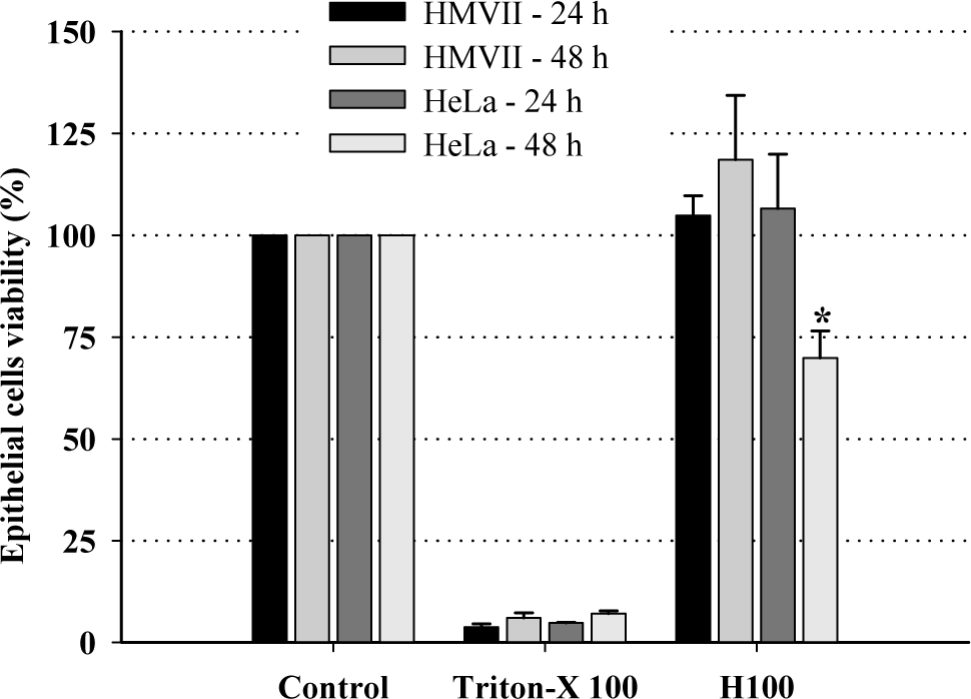
Effect of the H100 fraction on viability of human HMVII and HeLa lineages at 0.5 mg/mL. Controls represent cells in medium only, without H100. Data are the mean ± SD of at least three different experiments performed in triplicate. *P < 0.05 versus negative control.

The cytotoxicity of H100 to human cells was investigated for its potential against *T*. *vaginalis*. HMVII and HeLa cells were treated with 0.5 mg/mL H100 for 24 and 48h and then used for the MTT viability test. H100 was not cytotoxic against HMVII and HeLa lineages, except against HeLa after 48 h, when H100 presented low cytotoxicity (Fig 2). Importantly, this suggests that H100 has selective anti-trichomonads activity and does not affect host cells.

**Fig 3:**
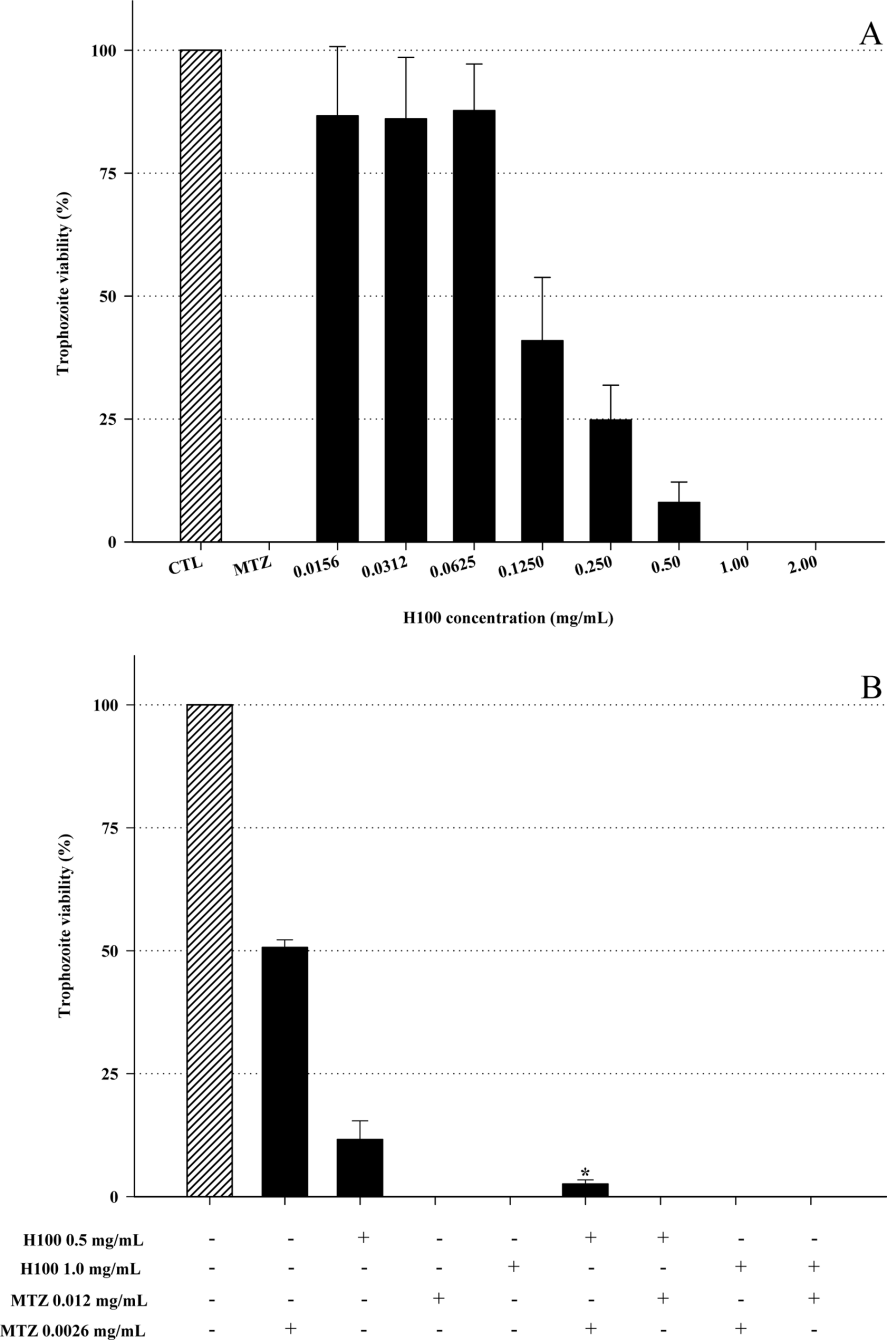
Combined effect of the H100 fraction and MTZ against *T*. *vaginalis* MTZ-resistant isolate. MIC determination of the H100 fraction against *T*. *vaginalis* TV-LACM2R (A); Synergic effect of H100 and MTZ against TV-LACM2 isolate (B). *P < 0.05 versus 0.0026 mg/mL MTZ treatment. Data are the mean ± SD of at least three different experiments (parasite suspensions) performed in triplicate.

Virulence and pathogenesis factors such as cytoadherence to epithelial cells and evasion of the immune system may differ between long-term grown and fresh-clinical isolates [28]. The H100 fraction was tested against eight *T*. *vaginalis* fresh-clinical isolates at 0.5 mg/mL. The results revealed that the H100 fraction was effective against all fresh-clinical isolates, with a reduction from 85 to 100% (Table 1). Another important result is that H100 enhanced the growth inhibitory effects of MTZ on MTZ-resistant isolate (TV-LACM2R). Resistance to MTZ is a growing concern, and drug resistance is expected to increase [2,3,29]. Alternatives to avoid this public health problem are necessary. The MIC of H100 against the TV-LACM2R isolate was 1.0 mg/mL (Fig 3A). The synergic effect of H100 and MTZ was observed when 0.5 mg/mL H100 (MIC/2) was associated with 0.0026 mg/mL MTZ (sub-lethal concentration). Alone, 0.0026 mg/mL MTZ reduced parasite viability by approximately 50%. In turn, in association with H100, a significant increase in the MTZ effect was achieved with diminished of *T*. *vaginalis* viability (97% reduction) (Fig 3B). Together, these results revealed that the H100 fraction is a promising alternative against *T*. *vaginalis* for both MTZ-sensitive and MTZ–resistant isolates, regardless of whether they were fresh clinical or long-term grown.

**Table 1:** Activity of H100 against different *T*. *vaginalis* isolates. The results are expressed as trophozoite viability (mean±SD) compared to untreated organisms (control).

*Trichomonas vaginalis* isolates	H100 (0.5 mg/mL)	
Control	100.00 ±	0.00
TVLAC-M2^a^	0.00 ±	0.00
TVLAC-M5	10.70 ±	1.51
TVLAC-M6	11.20 ±	1.24
TVLAC-M11	2.95 ±	1.31
TVLAC-M22	3.55 ±	0.51
TVLAC-M24	2.69 ±	1.09
TVLAC-H4	0.00 ±	0.00
TVLAC-H6	14.40 ±	2.53

^a^MTZ sensitive.

**Fig 4:**
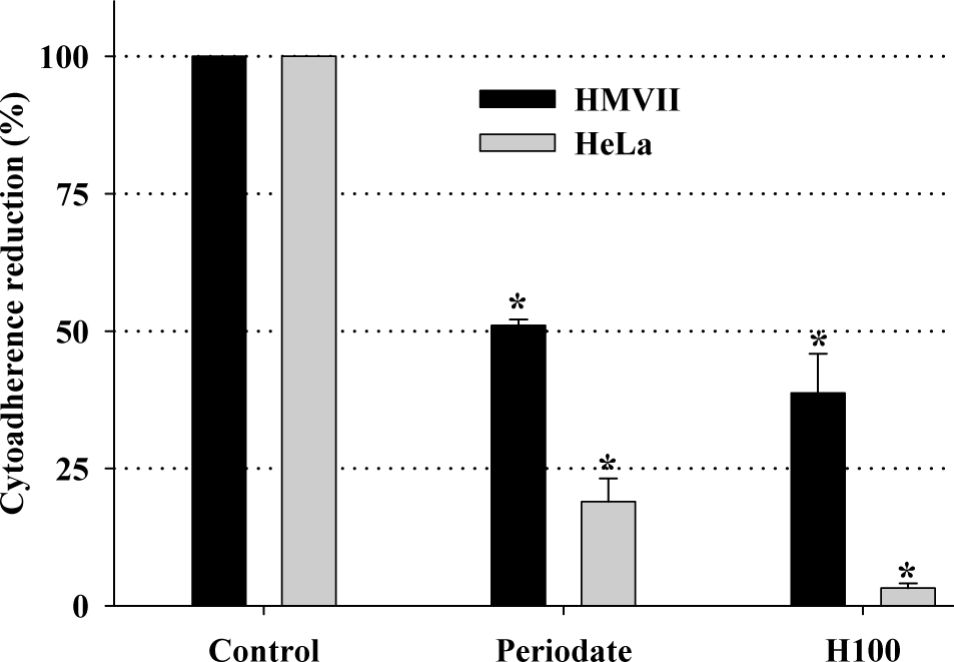
Effect of the H100 fraction on *T*. *vaginalis* TV-LACM6 isolate adhesion to HMVII and HeLa lineages. *treatments were statistically different from control. Data are the mean ± SD of at least three different experiments (parasite suspensions) performed in triplicate.

For extracellular pathogens such as *T*. *vaginalis*, the ability to adhere to the host cells is likely a determinant factor for pathogenesis. Cytoadherence is a complex mechanism that mainly involves surface proteins (adhesins) and glycoconjugates (lipophosphoglycan). In *T*. *vaginalis* cytoadherence is isolate-dependent, and the adherence level is directly related to the virulence of the isolate [28]. The *T*. *vaginalis* TV-LACM6 isolate is strongly adherent [30] and was selected to test the effect of H100 on cytoadherence. As expected, trophozoites treated with periodate (positive control), an agent that oxidizes surface polysaccharides such as lipophosphoglycan [31], reduced cytoadherence. Importantly, the adhesion of TV-LACM6 to HMVII and HeLa lineages was effectively reduced after H100 treatment (Fig 4), suggesting that the H100 fraction can induce a disruption in cytoadherence.

**Fig 5:**
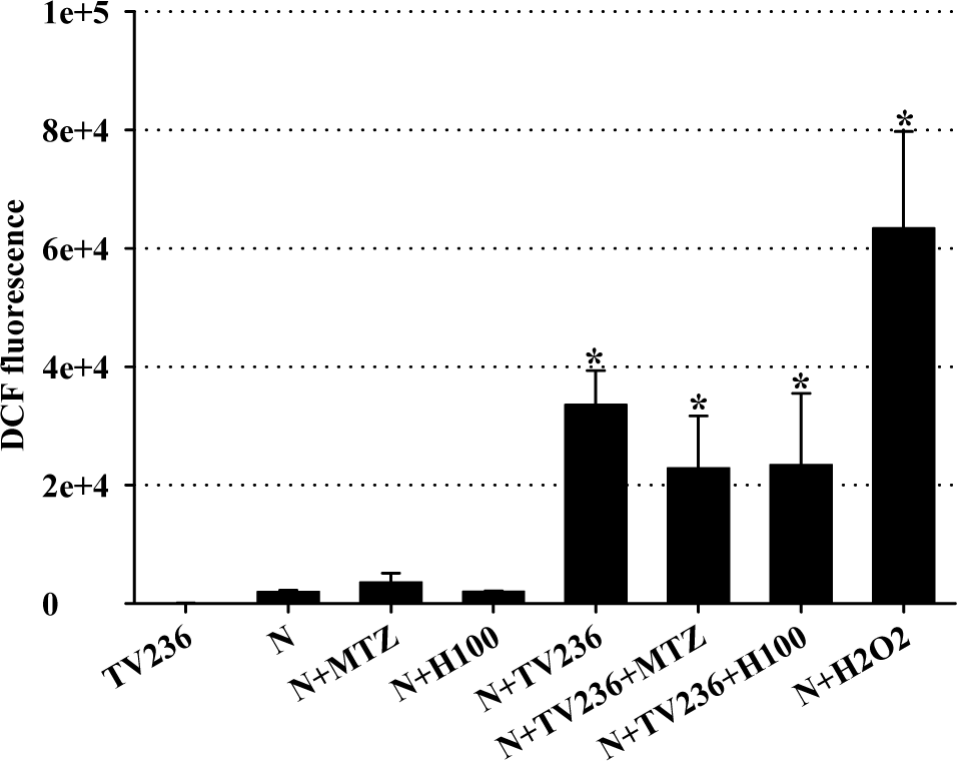
*Trichomonas vaginalis* promotes ROS generation in neutrophils. Co-incubation of human neutrophils with *T*. *vaginalis* treated with H100 and MTZ for 30 min and 10 μM DCF-DA for quantification of ROS production. *P < 0.05 versus neutrophils alone; 5.0 mM H_2_O_2_ is a positive control.

In addition, *T*. *vaginalis* triggers interleukin-8 (CXCL8) production from neutrophils at the infection site, and these immune cells are predominant in the vaginal discharge of patients with trichomoniasis [32]. Reactive oxygen species (ROS) have been identified as signaling messengers, and in neutrophils, they can eliminate phagocytosed microorganisms [24]. In this study, the effect of H100-treated *T*. *vaginalis* on ROS production by human neutrophils was evaluated. After 30 min of stimulation, untreated and H100-treated *T*. *vaginalis* induced significant ROS production compared to non-stimulated neutrophils (Fig 5). Notwithstanding, H100-treated trophozoites induced the same increase in ROS production as that in untreated trophozoites, indicating that the fraction could not affect ROS production by *T*. *vaginalis-*stimulated neutrophils. Organisms treated with MTZ were also unable to increase ROS production. These data indicate that the H100 fraction, as well as MTZ, did not play an immunomodulatory role in ROS production by neutrophils and are in agreement with other recent studies [33].

To evaluate the mechanism of death of *T*. *vaginalis* induced by H100, a set of experiments was performed. The hemolytic activity of H100 was evaluated, and after 1 h of incubation, hemolysis was not observed. In turn, H100-treated erythrocytes presented total lyses after 24 and 48 h of incubation (Table 2). Erythrocyte lysis is the most highlighted activity of saponins; lysis may occur due to the amphiphilic nature of the compounds that interact with lipids, leading to pore formation and membrane rupture [15–17]. Saponins are secondary metabolites with a wide spectrum of biological activities and can exhibit different activities depending on the chemical structures, which are mainly for monodesmosic and bidesmosic [34].

**Table 2:** Hemolytic activity of H100 at 0.5 mg/mL. The results were expressed as the percentage of erythrocytes lysis compared to positive control (Triton-X 100 0.2%).

	Hemolysis (%)					
	1 h		24 h		48 h	
Positive control	100.00 ±	0.00	100.00 ±	0.00	100.00 ±	0.00
Negative control	5.52 ±	0.90	8.16 ±	1.61	19.20 ±	7.25
H100	6.13 ±	1.21	98.40 ±	9.49	111.10 ±	7.97

The hypothesis that the H100 fraction may act on the parasite membrane was investigated by staining H100-treated organisms with DAPI and FLUTAX-2. FLUTAX-2 is an active Taxol fluorescent derivative and can bind and label the microtubules of intact trophozoites, allowing accurate observation using fluorescent microscopy [35]. Our results demonstrated that untreated organisms (control) were intact and that microtubules were stained with FLUTAX-2, while the nucleus was undamaged (DAPI-stained). Conversely, H100-treated trophozoites formed clusters of debris, which were stained with FLUTAX-2, indicating the presence of microtubules. They were not intact, and the teardrop classical morphology was disrupted but nuclear alterations were not observed (S3 Fig).

**Fig 6:**
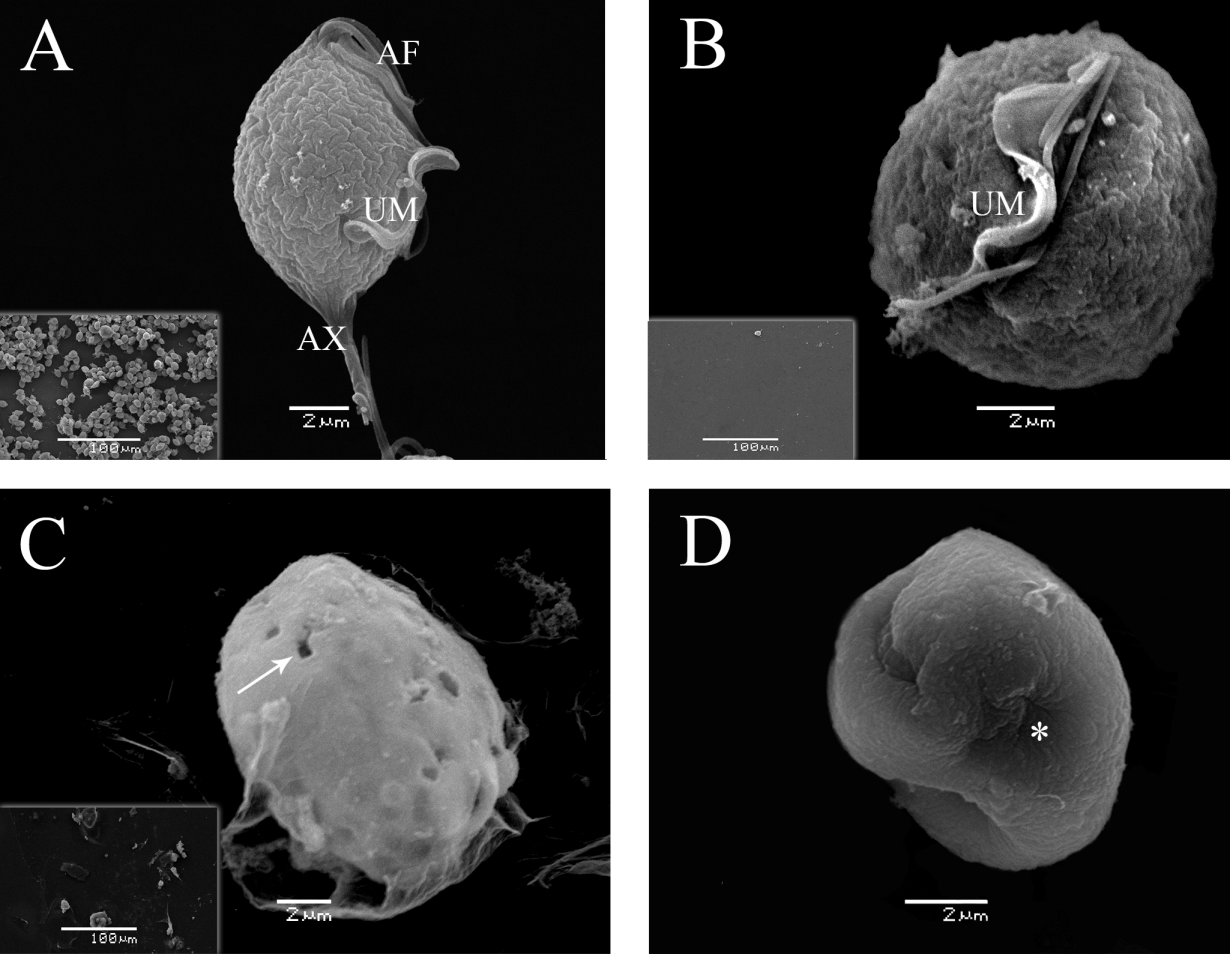
SEM of *T*. *vaginalis* after H100 fraction treatment. A classical untreated trophozoite displaying a tear-drop shape, four anterior flagella (AF), undulating membrane (UM) and axostyle (AX) (A); after 0.5 mg/mL H100 treatment for 4 h trophozoites displayed flagella and undulating membrane internalization (B), and after 24 h of treatment, organisms presented a wrinkled and fissured membrane (arrow) (C). Asterisks indicate complete flagella internalization (D).

**Fig 7:**
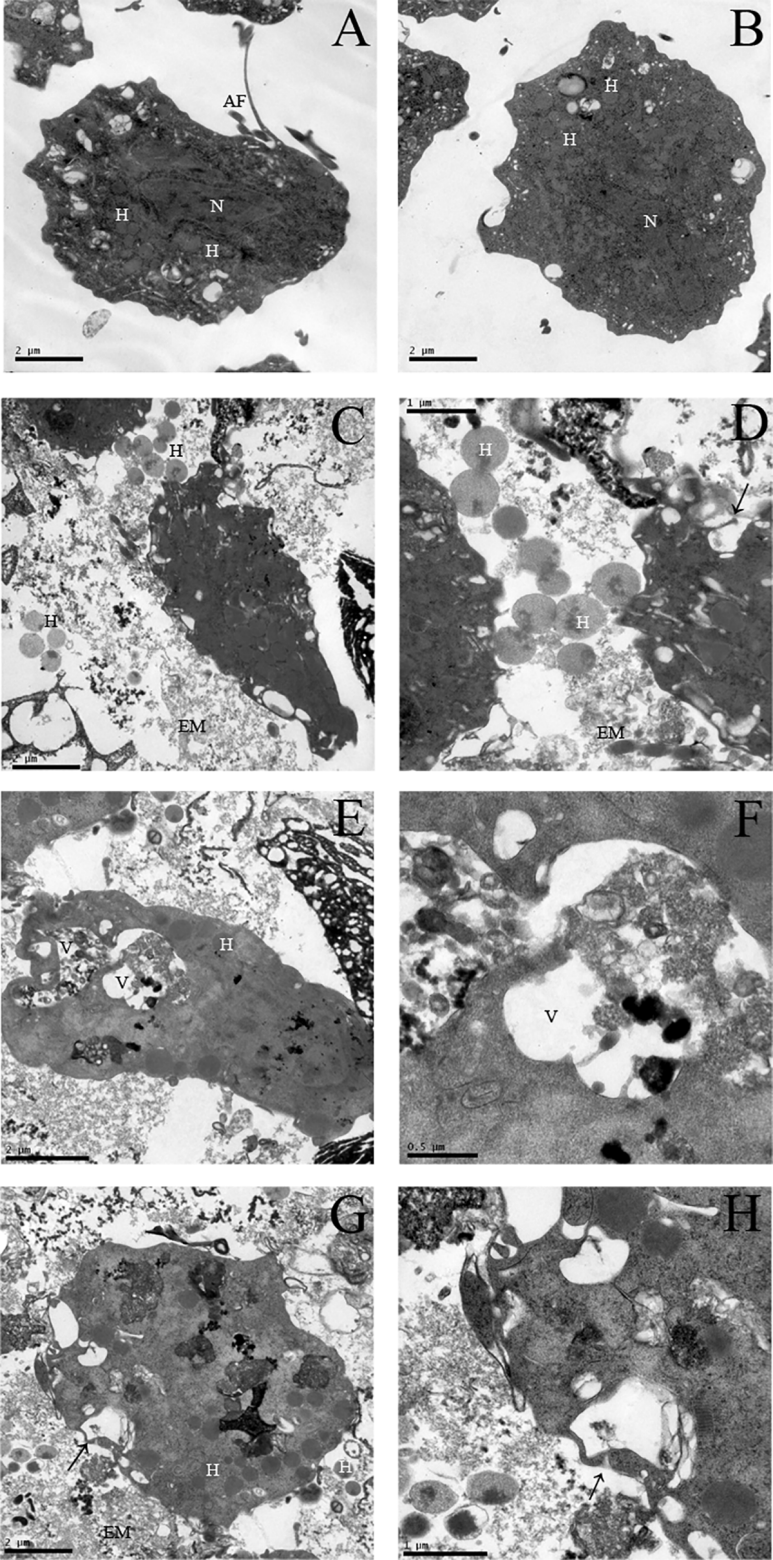
TEM of *T*. *vaginalis* under H100 fraction treatment. Control conditions presented one nucleus (N), hydrogenosomes (H) and anterior flagella (AF) (A-B); after 24 h of H100 treatment, trophozoites lost their classical morphology, hydrogenosomes were found in the extracellular medium (EM), and vacuoles (V), and arrows indicate membrane rupture (C-H).

Analyses of ultrastructural changes in *T*. *vaginalis* were performed using both SEM and TEM. The typical morphology of untreated *T*. *vaginalis* was observed using SEM, which is characterized as a teardrop shape, the presence of four anterior flagella, an undulating membrane and axostyle (Fig 6A). Using TEM, the expected intact morphology with one nucleus, hydrogenosomes and flagella was observed inside the trophozoites (Fig 7A and 7B). After treatment with H100 for 4 h, process of flagella internalization began (Fig 6B), but, pseudocysts were observed after 24 h (Fig 6C and 6D), suggesting time-dependent alteration as demonstrated after griseofulvin treatment [36]. Parasite number was reduced substantially by comparing untreated and treated organisms (Fig 6 insets). In addition, changes in the intracellular structure were also observed, such as intense cytoplasmic vacuolization, vacuoles with a membranous profile, membrane rupture and an intense distribution of hydrogenosomes in the extracellular milieu (Fig 7C–7H). Notably, after H100 treatment, the hydrogenosomes were not aligned with the axostyle but were spread out into the cytoplasm and extracellular milieu, indicating that the parasite membrane was affected and that all intracellular content was disturbed. These data corroborated the hemolytic assay results and fluorescence microscopy, as SEM and TEM analyses indicated that the H100 fraction mechanism of death occurred by damaging the parasite membrane.

**Fig 8:**
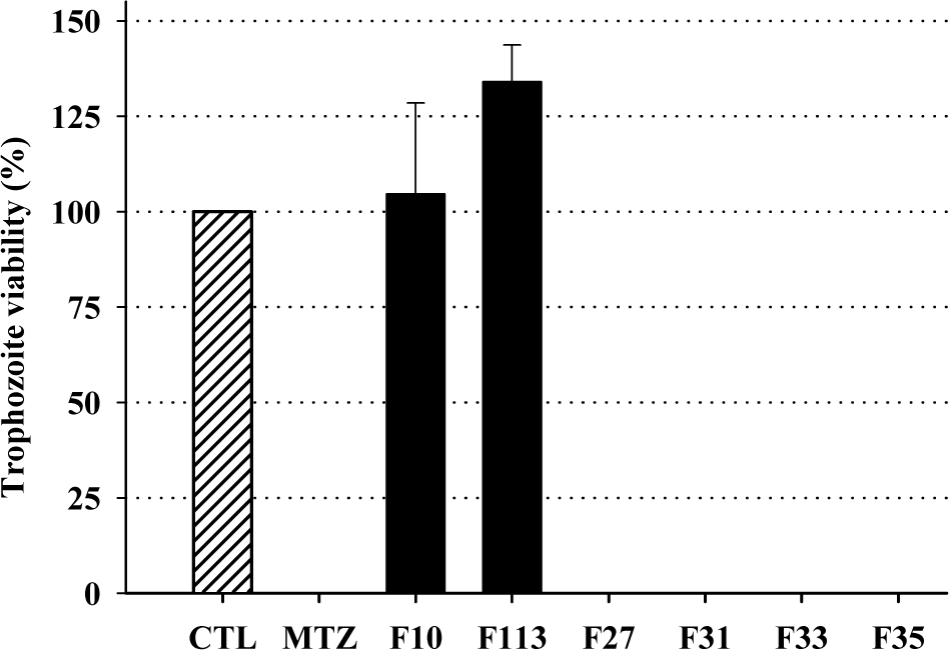
Anti-*T*. *vaginalis* activity of the purified fractions (0.5 mg/mL) from H100. Data are the mean ± SD of at least three different experiments (parasite suspensions) performed in triplicate.

To identify the compounds responsible for anti-*T*. *vaginalis* activity, the H100 fraction was purified by column chromatography yielding 130 fractions (F1-F130) and the activity was confirmed. The F10 and F113 fractions (S9 Fig) presented as the major components tannins and flavonoids. These compounds did not reduce parasite viability, and these findings agree with a previous study [37]. Conversely, 0.5 mg/mL F27, F31, F33, and F35 fractions totally disrupted the trophozoite viability (Fig 8). These fractions were constituted by identified bidesmosidic saponins (Table 3 and S10 Fig). These results corroborate our hypothesis that saponins were the active compounds responsible for *M*. *rufula* extract activity.

**Table 3:** Saponins identified from H100 by MALDI-LIFT-TOF.

MS (*m/z*) [M+Na]^+^	MS/MS (*m/z*)	MF	Saponin
689.4	527.3, 357.1, 203.0^pb^	C_36_H_58_O_11_	3-*O*-hex-protobassic acid
1099.5	689.4^pb^, 433.1, 301.1	C_52_H_84_O_23_	3-*O*-hex-28-*O*-(di-pent+deoxyhex)-protobassic acid
1231.6	689.4^pb^, 565.2, 433.1, 287.1	C_57_H_92_O_27_	3-*O*-hex-28-*O*-(tri-pent+deoxyhex)-protobassic acid
1245.6	689.4^pb^, 579.2, 447.1, 301.1	C_58_H_94_O_27_	3-*O*-hex-28-*O*-(di-pent+di-deoxyhex)-protobassic acid
1261.6	705.4, 689.4, 595.2^pb^, 579.2 463.1, 447.1, 317.1, 301.1	C_58_H_94_O_28_	3-*O*-hex-28-*O*-(di-pent+deoxyhex+hex)-protobassic acid 3-*O*-hex-28-*O*-(di-pent+di-deoxyhex)-16-hydroxyl-protobassic acid
1363.6	697.2^pb^, 689.4, 565.2, 433.1, 287.1	C_62_H_100_O_31_	3-*O*-hex-28-*O*-(tetra-pent+deoxyhex)-protobassic acid
1377.6	711.2^pb^, 689.4, 579.2, 447.1, 429.1, 301.1	C_63_H_102_O_31_	3-*O*-hex-28-*O*-(tri-pent+di-deoxyhex)-protobassic acid
1393.6	727.2^pb^, 689.4, 595.2, 463.1, 449.1, 317.1	C_63_H_102_O_32_	3-*O*-hex-28-*O*-(tri-pent+deoxyhex+hex)-protobassic acid
1407.6*	741.2^pb^, 689.4, 609.2, 463.1, 317.1	C_64_H_104_O_32_	3-*O*-β-D-glucopyranosyl-28-*O*-(α-L-rhamnopyranosyl-(1→3)-[β-D-glycopyranosyl-(1→4)]-β-D-xylopyranosyl-(1→4)-α-L-rhamnopyranosyl-(1→2)-α-L-arabinopyranosyl)-protobassic acid
1423.6	757.2, 741.2^pb^, 705.4, 689.4, 609.2, 595.2, 463.1, 317.1	C_64_H_104_O_33_	3-*O*-hex-28-*O*-(di-pent+deoxyhex+di-hex) -protobassic acid 3-*O*-hex-28-*O*-(di-pent+di-deoxyhex+hex) -16-hydroxyl-protobassic acid

MF: molecular formula.

*: isolated saponins.

^pb^: peak base; hex: hexosyl, dipent: pentosyl; deoxyhex: deoxyhexosyl.

**Fig 9:**
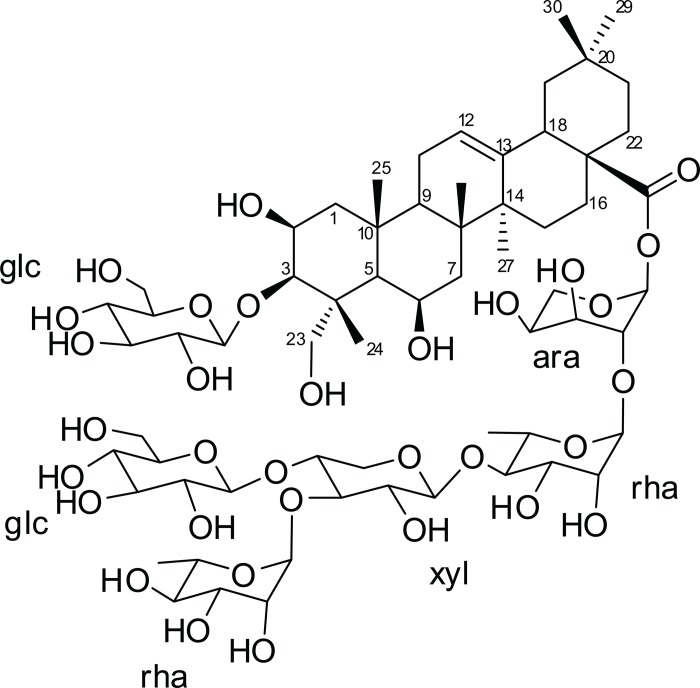
Chemical structure of saponin 1 isolated from *Manilkara rufula*.

The structure of isolated saponin 1 (F23-F25) was identified as Mi-saponin C (Fig 9) using 1D and 2D NMR data and was confirmed using high resolution ESI mass spectrometry (S7 Fig). All spectrometric data and discussions are described in S1 and S2 Texts.

**Fig 10:**
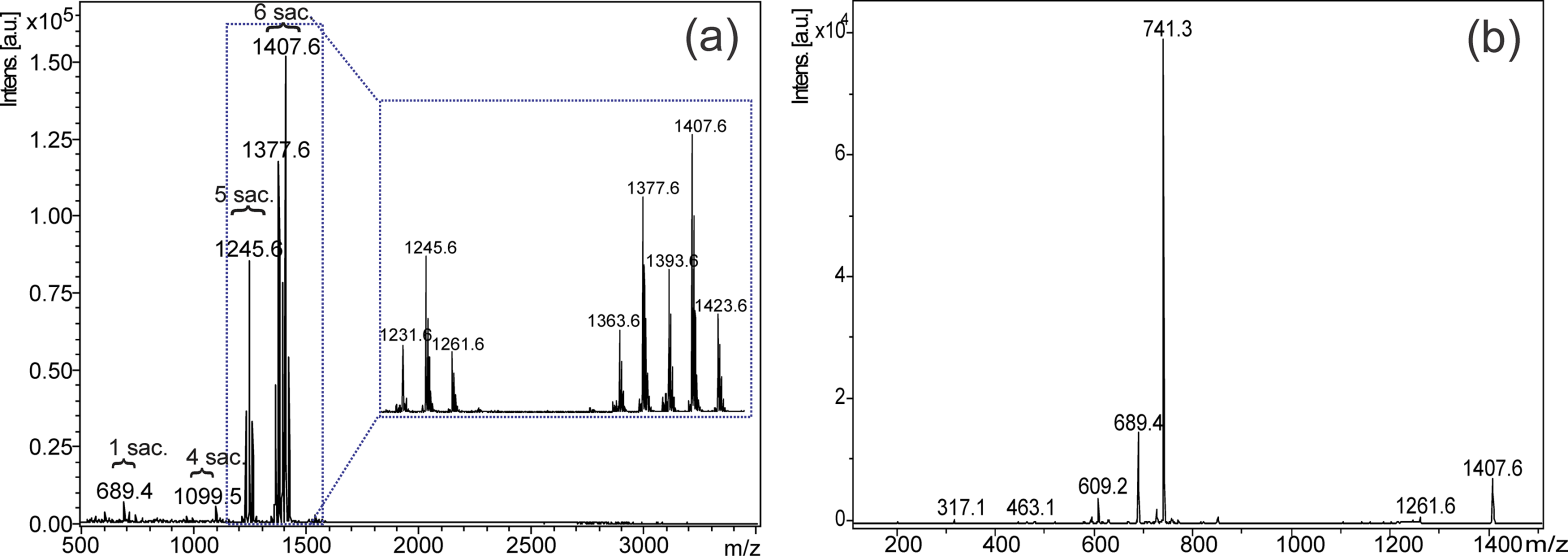
MALDI analyses of the H100 fraction. MALDI-MS of the H100 fraction (A) and MALDI-MS/MS spectra of the *m/z* 1407 [M+Na]^+^ ion relative to saponin 1 (B) (sac: saccharide).

**Fig 11:**
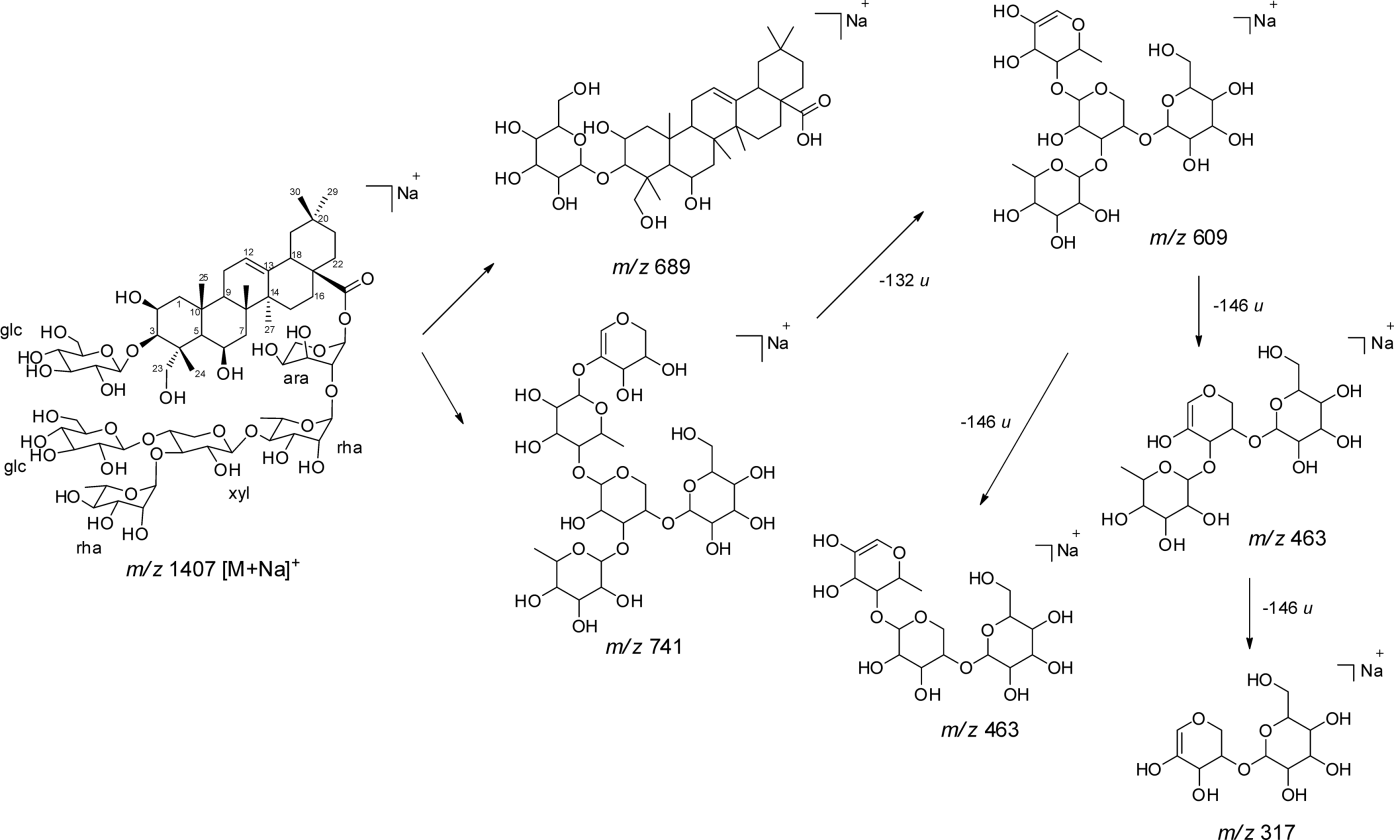
Main fragment ions of saponin 1 obtained by MALDI-LIFT TOF.

The H100 fraction was also analyzed by LC-ESI-MS to determine the presence of isobaric saponins (S8 Fig), but the number of saponins identified by MALDI was higher than ESI, as reported by Chapagain and Wiesman [38]. For this reason, we described in MALDI analyses in detail (Fig 10). First, the fragmentation of Mi-saponin C (1) by MALDI-LIFT-TOF (Fractions F23-F25) (Figs 10A and 11) was evaluated, and this knowledge was applied to identify other saponins from the H100 fraction. MALDI-LIFT-TOF analyses mainly showed fragment ions from saccharide chains, facilitating the interpretation and elucidation.

The MS/MS of Mi-saponin C (1) (*m/z* 1407 [M+Na]^+^) yielded fragment ions at *m/z* 741, 689, 609, 463 and 317 (Fig 10B), showing a strong predominance of the fragments produced from the glycosidic moiety, similar to those observed for saponins, bile acids and conjugated steroids [39]. The main fragment ions were observed at *m/z* 689 [M+Na-saccharide ester chain]^+^ and 741 [M+Na-(aglycone+glucoside)]^+^, which are related to 3-*O*-glucopyranosyl-protobassic acid and the saccharide ester chain at C-28 (Fig 11), respectively; these confirmed both parts of the saponin. Subsequently, fragment ions produced by consecutive losses of sugars from *m/z* 741 confirmed the saccharide composition in the portion of saccharide ester chain, such as the ions at *m/z* 609, 463 and 317, which are formed by the loss of a pentose (arabinose), and then two deoxyhexoses (rhamnose). The fragmentation observed by MALDI-LIFT-TOF in our study was similar to those reported for MALDI-Post Source Decay (PSD) fragmentation, and diagnostic and sufficient ions for the identification of saponins were visualized [39,40].

Similar fragmentation was observed for the other saponins presented in the H100 fraction, which were identified. From the MALDI-MS spectrum of the fraction, saponins with four (*m/z* 1099 [M+Na]^+^), five (*m/z* 1231, 1245 and 1261) and six saccharides (*m/z* 1363, 1393, 1407 and 1423) were detected; in addition, the protobassic acid linked to one sugar (*m/z* 689 [M+Na]^+^) was also detected (Fig 10B and Table 3). The fragment ion at *m/z* 689 was observed for all saponins and confirmed the 3-*O*-hexoside protobassic acid moiety; in addition, the saccharide ester chain was determined using the fragment ions yielded the loss of 3-*O*-hexoside protobassic acid (666 *u*), where the sodium ion is located.

The ion *m/z* 1261 produced the fragment ions *m/z* 689 and also the ion *m/z* 705, suggesting isobaric saponin structures. The ion *m/z* 705 is related to the 16-hydroxyl- protobassic acid, as confirmed by the fragment from the saccharide ester moiety (*m/z* 579) to determine the two structural moieties. This triterpenic aglycone was already identified in the saponins of *M*. *hexandra* [41]. Thus, the sugar losses from *m/z* 579 confirmed these units, such as *m/z* 447 and 301, that were produced from the loss of a pentose and a deoxyhexose, respectively. In the same way, twelve bidesmosic saponins were identified from the H100 fraction (Table 3), and their fragmentation pathways are compatible which the fragmentation of other saponins reported in the literature [10,38–40,42,43].

Saponins have showen a potent anti-trichomonas activity, but are still underexplored, and few studies have reported this application. This study demonstrated the potent activity of a bidesmosic saponin from *Manilkara rufula* against *T*. *vaginalis*. The activity of this saponin is higher than that presented by a fraction of bidesmosic saponins from *Ilex paraguariensis*, as demonstrated in previous studies [15,44]. In addition, the fraction of monodesmosic saponins from *Sapindus* sp. [20], *Hedera colchica* [18] and *Buddleja madagascariensis* [19]also exhibited high anti-trichomonas activity, showing that the relationship between the strutucture of saponins and their anti-parasitic activity is still not clear. Together, the results presented in this study demonstrated the potential of *Manilkara rufula* bidesmosic saponin against *T*. *vaginalis*, inducing parasite death due to profound membrane damage.

## Supporting information

S1 Text Chemical structure elucidation of the isolated saponin 1. (PDF)

S2 Text Identification of constituents from the H100 fraction by LC-MS and MALDI-MS. (PDF)

S1 Fig Parasite viability determination using FDA-PI cytometry. Dot plots of parasite viability treated or untreated with H100 label with FDA-PI.
(PDF)

S2 Fig Quantification of parasite viability from the flow cytometry assay. (PDF)

S3 Fig Confocal microscopy of *T*. *vaginalis* stained with 5.0 μg/mL DAPI and 1.0 μM FLUTAX-2. Control showing classical teardrop shape and four anterior flagella and axostyle (A); H100-treated trophozoites after 4 (B) and 24 h (C) demonstrating striking membrane alterations and clusters formation. Magnification 60x.
(PDF)

S4 Fig Chemical structure of Mi-saponin C (1) isolated and the main HMBC correlations. (PDF)

S5 Fig HMQC spectrum of saponin 1 (CD_3_OD, 500 MHz). (PDF)

S6 Fig HMBC spectrum of saponin 1 (CD_3_OD, 500 MHz). (PDF)

S7 Fig HR-ESI MS of saponin 1 (negative ion mode). (PDF)

S8 Fig LC-ESI MS of fraction H100 (negative ion mode). (PDF)

S9 Fig MALDI-MS of fractions 10 (F10) and 113 (F113) (positive ion mode). (PDF)

S10 Fig MALDI-MS of fractions 27 (F27), 31 (F31), 33 (F33) and 35 (F35) (positive ion mode). (PDF)

S1 Table ^1^H and ^13^C NMR spectroscopic data of saponin 1 (500 MHz, CD_3_OD). (PDF)
