# Supplementary material for: Trichomonicidal and parasite membrane damaging activity of bidesmosic saponins from Manilkara rufula
Source: PLoS One. 2017 Nov 30;12(11):e0188531. doi: 10.1371/journal.pone.0188531 (PMC5708768; doi:10.1371/journal.pone.0188531)
Supplement: S1 Text — (PDF) [file pone.0188531.s001.pdf]

S1 Text.

The ^{1}H NMR spectrum of saponin 1 revealed six signals relative to methyl groups at δ 0.90 (*s*), 0.94 (*s*), 1.05 (*s*), 1.13 (*s*), 1.31 (*s*) and 1.61 (*s*) (S1 Table) that correlated to the carbon signals at δ 33.6 (C-29), 24.2 (C-30), 18.8 (C-26), 26.4 (C-27), 16.4 (C-24) and 19.2 (C-25) in the HMQC spectrum (S5 Fig). The attributions of signals were determined based on the HMBC correlations (S4 and S6 Fig), such as the correlations of H/C-29 with signals of 30 and 22, H/C-26 with 14 and 8 and H/C-25 with 1 and 9 positions. The carbons at δ 83.8 (C-3) and 177.9 (C-28) indicates the presence of two saccharide chains attaching the hydroxyl at C-3 and in C-28 esterifying the acid, which were confirmed by the H-C correlations of the anomeric hydrogens observed in the HMBC. In addition, hydroxylations were established in other aglycone positions from the hydrogens δ 4.32 (*m*, H-2), 4.46 (*m*, H-6) and 3.42/3.72 (H-23), as well as the presence of one double bond at C-12 was also determined from the hydrogen at δ 5.35(*t*) and the carbons δ 124.4 (C-12) and 144.4 (C-13). Thus the data confirmed the aglycone protobassic acid, and they are in agreement with those reported [1], besides this aglycone was already reported in *Manilkara* species [2].

The six anomeric hydrogens were observed in the region from 4.2 to 5.7 ppm, suggesting the presence of six sugars in the structure. Their coupling constant (*J*) were important to determine the anomeric configuration, for example, 7-8 Hz and 1-4 Hz for β- and α-configuration in the pyranosides, respectively [1,2]. The signals of each sugar were established by the H-H correlations observed in the COSY and TOCSY 2D spectra. The signals at δ 4.42 (*d*, J= 7.7 Hz) and 4.28 (*d*, *J* = 7.5 Hz), relative to the carbons at δ 105.4 and 103.9 observed from HMQC (S5 Fig), together with all their ^{1}H and ^{13}C NMR data confirmed the identity of β-glucose. The same strategy was applied to elucidate the other saccharides with the anomeric hydrogens at δ 4.52 (*d*, *J*= 7.6 Hz), 5.09 (*d*, *J*= 1.2Hz), 5.17 (*d*, *J*= 1.4Hz) and 5.62 (*d*, *J*= 4.0 Hz), which were identified as α-xylose, α-rhamnose, α-rhamnose and α-arabinose, respectively, and these data were compatible to the data reported in the literature.[1,2] The linkage position of sugars were determined by the C-H correlations observed from the HMBC spectrum (S4 and S6 Fig), for example, the correlations of δ 5.62 (H-1 of α-arabinose) and 4.42 (H-1 of β-glucose) with the aglycone carbons δ 83.8 (C-3) and 177.9 (C-28). Thus the isolated saponin 1 as 3-*O*-β-D-glucopyranosyl-28-*O*-(α-L-rhamnopyranosyl-(1→3)-[β-D-glycopyranosyl-(1→4)]-β-D-xylopyranosyl-(1→4)-α-L-rhamnopyranosyl-(1→2)-α-L-arabinopyranosyl)-protobassic acid or Mi-saponin C. The ion at *m/z* 1383.6423 $[M-H]^-$ was observed in the high resolution mass spectrum of saponin 1 (S7 Fig), confirming the structure proposed ($C_{64}H_{103}O_{32}^-$, calc. 1383.6438, error 1.0 pmm).

Therefore, the isolated saponin was identified as Mi-saponin C, which was described from species *Planchonella* and *Madhuca longifolia* and whose spectral data are compatible with those obtained in the present study.

References

1. Eskander J, Lavaud C, Abdel-Khalik SM, Soliman HS, Mahmoud, II, Long C. Saponins from the leaves of *Mimusops laurifolia*. J Nat Prod. 2005; 68: 832-841.
2. Eskander J, Lavaud C, Pouny I, Soliman HS, Abdel-Khalik SM, Mahmoud II. Saponins from the seeds of *Mimusops laurifolia*. Phytochemistry. 2006; 67: 1793-1799.
