# Supplementary material for: Trichomonicidal and parasite membrane damaging activity of bidesmosic saponins from Manilkara rufula
Source: PLoS One. 2017 Nov 30;12(11):e0188531. doi: 10.1371/journal.pone.0188531 (PMC5708768; doi:10.1371/journal.pone.0188531)
Supplement: S2 Text — (PDF) [file pone.0188531.s002.pdf]

## S2 Text.

As described in the manuscript, the H100 fraction was also analysed by LC-ESI-MS to determine the presence of isobaric saponins (S8 Fig). The saponins were identified by their fragmentation pathway by comparing data of Mi-saponin C (1), obtained by MALDI-LIFT TOF. By the structural determinations, ten saponins were identified (information in the manuscript). From H100, 130 fractions were obtained. The F10 and F113 fractions were analysed by MALDI-TOF (S9 Fig) and saponins were not detected. In addition, the F27, F31, F33 and F35 were also analysed by MALDI-TOF (S10 Fig), and bidesmosidic saponins were identified (chemical elucidation in the manuscript).
