# Supplementary figures and images for: Trichomonicidal and parasite membrane damaging activity of bidesmosic saponins from Manilkara rufula
Source: PLoS One. 2017 Nov 30;12(11):e0188531. doi: 10.1371/journal.pone.0188531 (PMC5708768; doi:10.1371/journal.pone.0188531)

# Results and discussion

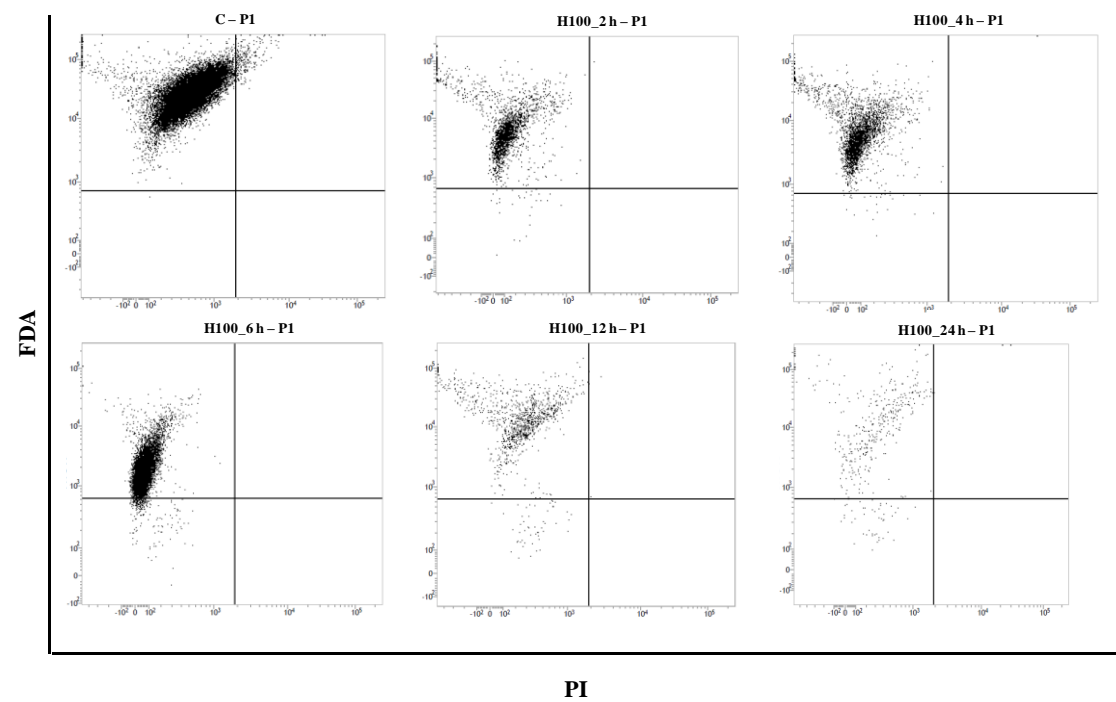

S1 Fig.

Supplement: S1 Fig — Dot plots of parasite viability treated or untreated with H100 label with FDA-PI. (PDF) [file pone.0188531.s003.pdf]

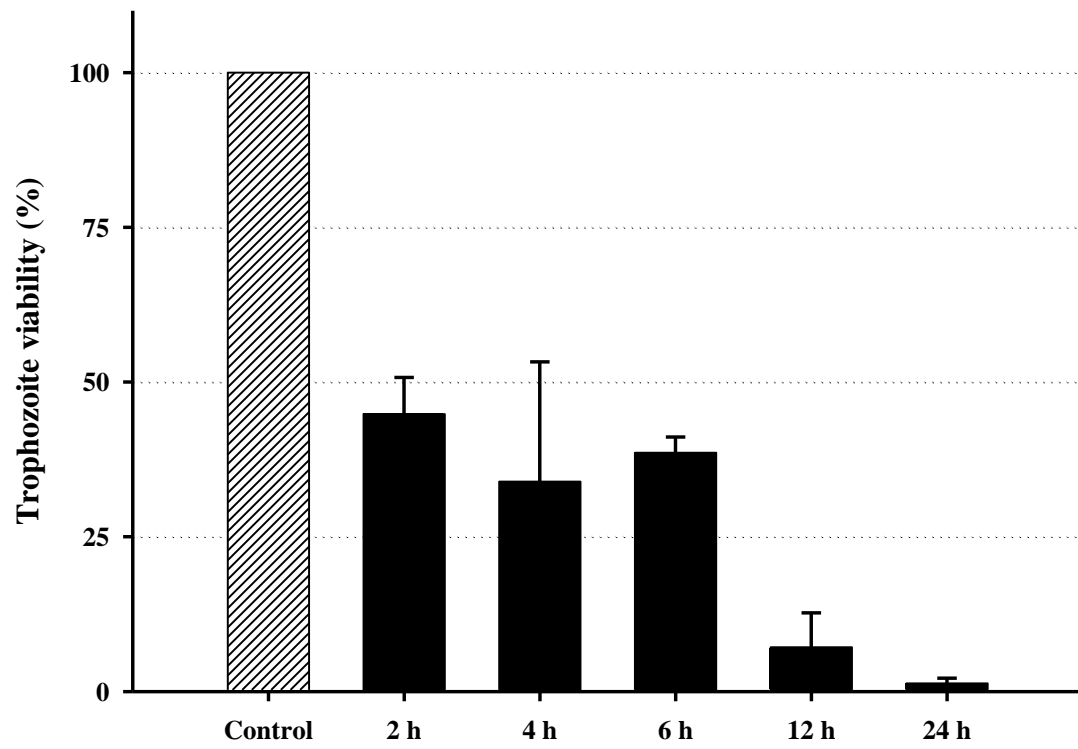

**S2 Fig.**

Supplement: S2 Fig — (PDF) [file pone.0188531.s004.pdf]

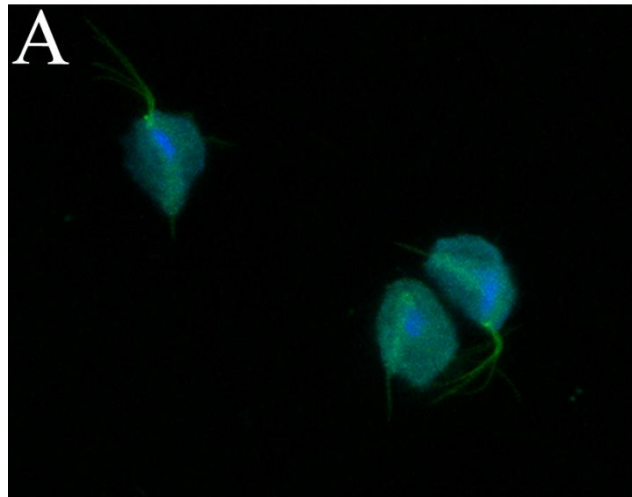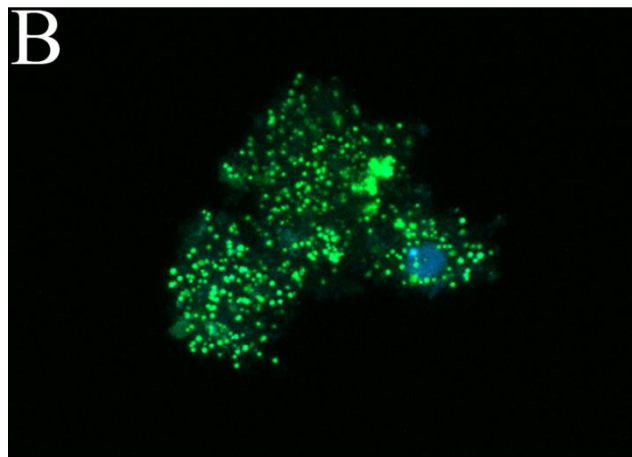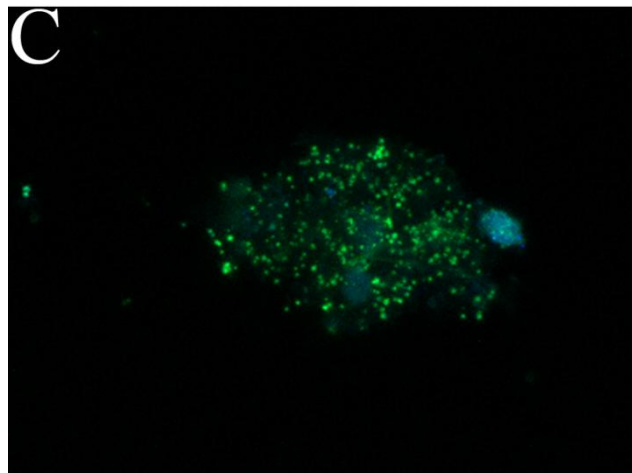

**S3 Fig.**

Supplement: S3 Fig — Control showing classical teardrop shape and four anterior flagella and axostyle (A); H100-treated trophozoites after 4 (B) and 24 h (C) demonstrating striking membrane alterations and clusters formation. Magnification 60x. (PDF) [file pone.0188531.s005.pdf]

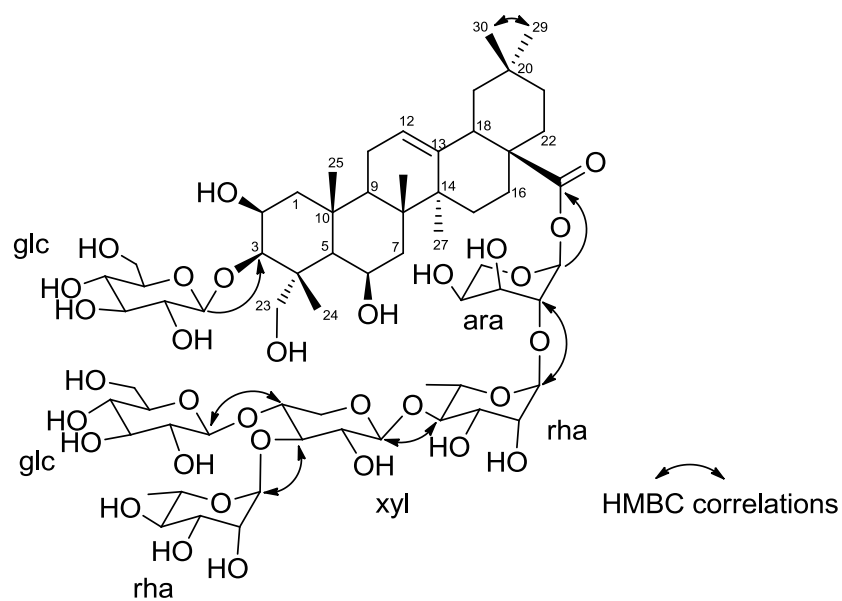

**S4 Fig.**

Supplement: S4 Fig — (PDF) [file pone.0188531.s006.pdf]

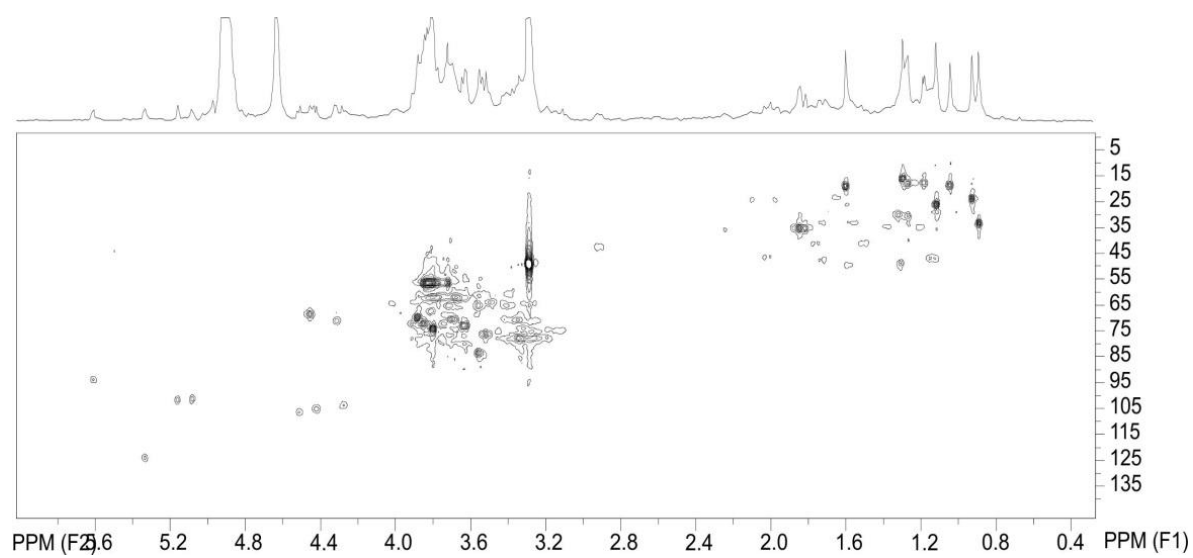

**S5 Fig.**

Supplement: S5 Fig — (PDF) [file pone.0188531.s007.pdf]

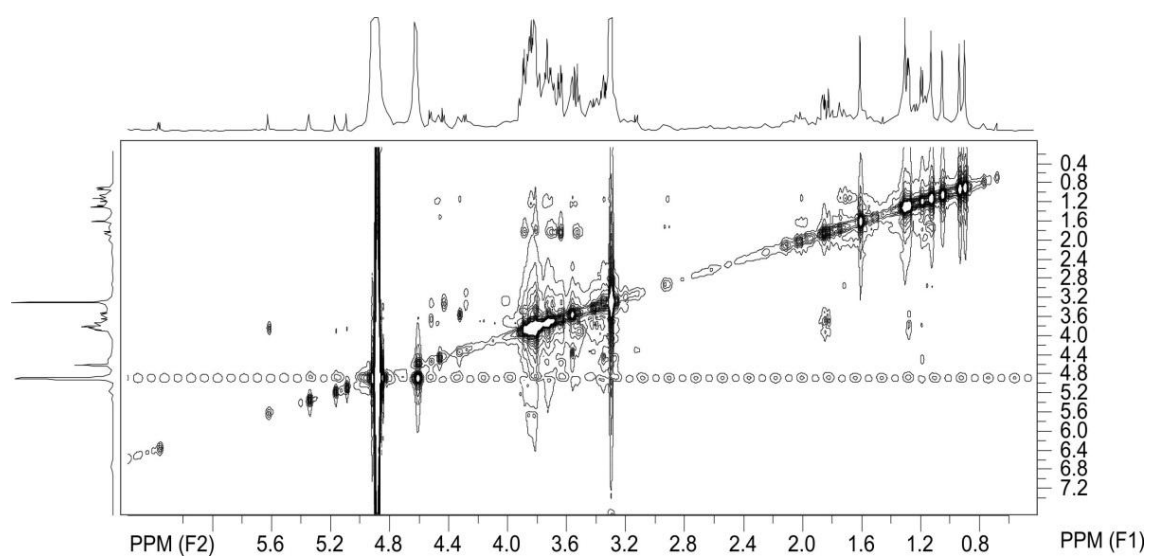

**S6 Fig.**

Supplement: S6 Fig — (PDF) [file pone.0188531.s008.pdf]

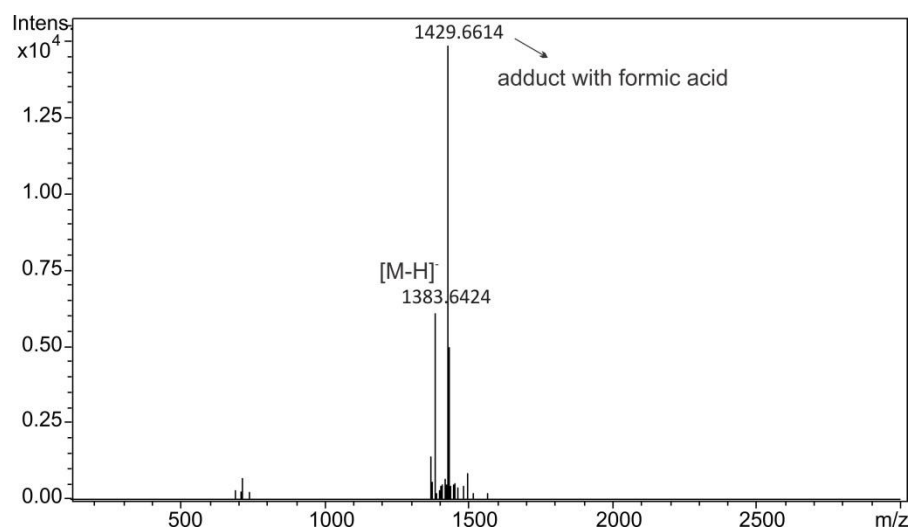

**S7 Fig.**

Supplement: S7 Fig — (PDF) [file pone.0188531.s009.pdf]

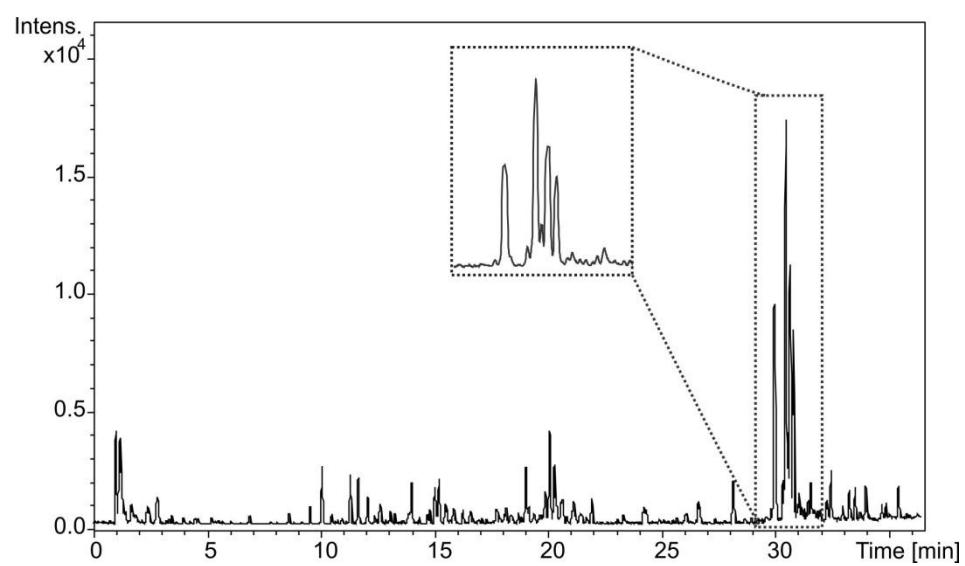

**S8 Fig.**

Supplement: S8 Fig — (PDF) [file pone.0188531.s010.pdf]

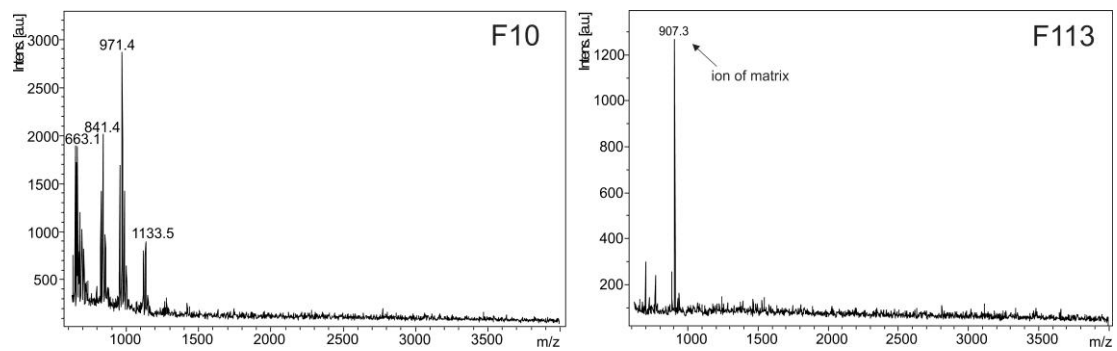

**S9 Fig.**

Supplement: S9 Fig — (PDF) [file pone.0188531.s011.pdf]

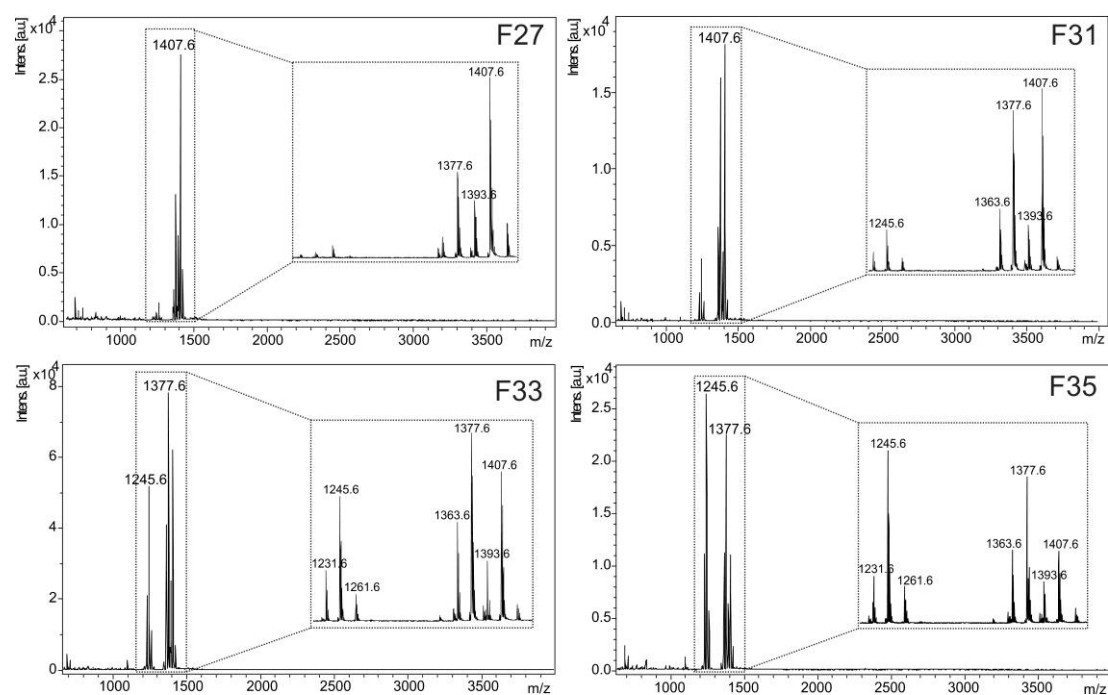

**S10 Fig.**

Supplement: S10 Fig — (PDF) [file pone.0188531.s012.pdf]
