# Supplementary material for: Trichomonicidal and parasite membrane damaging activity of bidesmosic saponins from Manilkara rufula
Source: PLoS One. 2017 Nov 30;12(11):e0188531. doi: 10.1371/journal.pone.0188531 (PMC5708768; doi:10.1371/journal.pone.0188531)
Supplement: S1 Table — (PDF) [file pone.0188531.s013.pdf]

S1 Table.

H/C	δ_C	δ_H (*J* in Hz)	H/C	δ_C	δ_H (*J* in Hz)
1	46.8	1.13$^{\theta}$	3-*O*-β-glucose		
		2.03, *dd* (2.0, 14.0)	1	105.4	4.42, *d* (7.7)
2	71.3	4.32, *m*	2	75.6	3.27$^{\theta}$
3	83.8	3.55$^{\theta}$	3	78.2	3.33, *m*
4	44.3	-	4	71.2	3.35, *dd* (7.0, 8.9)
5	49.2	1.30$^{\theta}$	5	78.1	3.30$^{\theta}$
6	68.7	4.46, *m* ($W_{1/2}$ 9.8)	6	62.4	3.70, *dd* (4.8, 11.5)
					3.81, *dd* (1.7, 11.5)
7	41.4	1.50, br *d* (14.0)	28-*O*-α-arabinose (ester)		
		1.79$^{\theta}$	1	94.2	5.62, *d* (4.0)
8	40.0	-	2	75.6	3.81$^{\theta}$
9	49.7	1.59$^{\theta}$	3	70.7	3.89, *t* (3.5)
10	37.3	-	4	67.7	3.83, *m*
11	24.5	1.97, *m*	5	64.2	3.53, *t* (9.0)
		2.09, *m*			3.92$^{\theta}$
12	124.4	5.35, *t* (3.0)	α-rhamnose (at C-2 Ara)		
13	144.4	-	1	101.5	5.09, *d* (1.2)
14	43.8	-	2	72.5	3.91$^{\theta}$
15	29.4	1.30$^{\theta}$	3	72.5	3.88$^{\theta}$
16	24.0	1.64, br *d* (15.3)	4	83.5	3.58, *t* (8.7)
17	48.7	-	5	69.1	3.74$^{\theta}$
18	42.8	2.92, *dd* (3.4, 13.4)	6	18.1	1.28, *d* (6.0)
19	47.2	1.14, br *d* (12.2)	α-xylose (at C-4 Rham)		
		1.75, br *d* (14.0)	1	106.6	4.52, *d* (7.6)
20	31.7	-	2	76.3	3.53, *t* (9.0)
21	35.3	1.23, *m*	3	80.1	3.65$^{\theta}$
		1.81, *td* (3.0, 13.6)	4	74.4	3.81$^{\theta}$
22	33.8	1.57, *m*	5	65.1	3.57, *m*
		1.73, *m*			3.73$^{\theta}$
23	65.6	3.42, *d* (11.2)	α-rhamnose (at C-3 Xyl)		
		3.72$^{\theta}$	1	102.0	5.17, *d* (1.4)
24	16.4	1.31 (*s*)	2	72.5	3.91, *t* (1.4, 3.4)
25	19.2	1.61 (*s*)	3	72.7	3.74, *m*
26	18.8	1.05 (*s*)	4	74.0	3.63$^{\theta}$
27	26.4	1.13 (*s*)	5	71.0	4.30, *m*
28	177.9	-	6	18.0	1.19, *d* (6.1)
29	33.6	0.90 (*s*)	3-*O*-β-glucose (at C-4 Xyl)		
30	24.2	0.94 (*s*)	1	103.9	4.28, *d* (7.5)
--	--	--	2	75.2	3.12, *dd* (7.5, 9.0)
--	--	--	3	78.2	3.30
--	--	--	4	72.3	3.74$^{\theta}$
--	--	--	5	78.2	3.37
--	--	--	6	62.3	3.57, *m*
					3.88, *m*
